# Patients with an unexplained microsatellite instable tumour have a low risk of familial cancer

**DOI:** 10.1038/sj.bjc.6603754

**Published:** 2007-04-24

**Authors:** L I H Overbeek, C M Kets, K M Hebeda, D Bodmer, E van der Looij, R Willems, M Goossens, N Arts, H G Brunner, J H J M van Krieken, N Hoogerbrugge, M J L Ligtenberg

**Affiliations:** 1 Department of Human Genetics 849, Radboud University Nijmegen Medical Centre, PO Box 9101, 6500 HB Nijmegen, The Netherlands; 2 Department of Pathology, Radboud University Nijmegen Medical Centre, PO Box 9101, 6500 HB Nijmegen, The Netherlands

**Keywords:** colorectal neoplasms, hereditary nonpolyposis, microsatellite instability, DNA mismatch repair, DNA methylation

## Abstract

The cancer risk is unknown for those families in which a microsatellite instable tumour is neither explained by *MLH1* promoter methylation nor by a germline mutation in a mismatch repair (MMR) gene. Such information is essential for genetic counselling. Families suspected of Lynch syndrome (*n*=614) were analysed for microsatellite instability, *MLH1* promoter methylation and/or germline mutations in *MLH1*, *MSH2*, *MSH6*, and *PMS2.* Characteristics of the 76 families with a germline mutation (24 *MLH1*, 2 *PMS2*, 32 *MSH2*, and 18 *MSH6*) were compared with those of 18 families with an unexplained microsatellite instable tumour. The mean age at diagnosis of the index patients in both groups was comparable at 44 years. Immunohistochemistry confirmed the loss of an MMR protein. Together this suggests germline inactivation of a known gene. The Amsterdam II criteria were fulfilled in 50/75 families (66%) that carried a germline mutation in an MMR gene and in only 2/18 families (11%) with an unexplained microsatellite instable tumour (*P*<0.0001). Current diagnostic strategies can detect almost all highly penetrant MMR gene mutations. Patients with an as yet unexplained microsatellite instable tumour likely carry a different type of mutation that confers a lower risk of cancer for relatives.

Lynch syndrome (Hereditary NonPolyposis Colorectal Cancer (HNPCC)) accounts for about 5% of colorectal cancers and is caused by a germline mutation in one of the mismatch repair (MMR) genes (Aaltonen *et al*, 1998; Cunningham *et al*, 2001; Lynch and de la Chapelle, 2003; Hampel *et al*, 2005; Barnetson *et al*, 2006). Known MMR genes causing Lynch syndrome are *MLH1*, *PMS2*, *MSH2*, and *MSH6*. Typical Lynch syndrome families show autosomal dominant predisposition to a number of cancers of which colorectal cancer is the most important. Conversely, over 90% of colorectal cancers of Lynch syndrome patients have a defect in the MMR system (Lynch and de la Chapelle, 2003). Failure of the DNA MMR system causes microsatellite instability (MSI) in tumours.

MSI reflects either the presence of a germline mutation in the MMR system or somatic hypermethylation of the promoter region of the *MLH1* gene (Cunningham *et al*, 2001). Patients with a tumour with MSI and somatic hypermethylation of the *MLH1* promoter rarely carry a germline mutation in the MMR system, although rare exceptions have been reported. A few families have been described in which Lynch syndrome patients display hypermethylation of the *MLH1* promoter in tumour as well as in non-tumour tissue (Gazzoli *et al*, 2002; Miyakura *et al*, 2004; Suter *et al*, 2004; Hitchins *et al*, 2005; Valle *et al*, 2007a). In addition, a family was recently described in which the susceptibility to tumours is caused by germline methylation of the *MSH2* promoter (Chan *et al*, 2006). Taken together, hypermethylation of the *MLH1* promoter indicates a very low likelihood that Lynch syndrome is the cause of the MSI (Samowitz *et al*, 2005; Weisenberger *et al*, 2006). Such patients can be offered less stringent surveillance programs (Lindor *et al*, 2005; Dove-Edwin *et al*, 2006; Valle *et al*, 2007b).

Little is known about the cancer risk in those families in which MSI is detected in a tumour, but where the MSI can be explained neither by hypermethylation of the *MLH1* promoter nor by a germline mutation in an MMR gene. Such information is essential for genetic counselling.

In the present study, we examined patients with a tumour that indicated possible Lynch syndrome for germline mutations in the MMR genes *MLH1*, *MSH2*, *MSH6*, and *PMS2*. In addition, we tested tumour DNA for hypermethylation of the *MLH1* promoter. Most of these patients were preselected by MSI analysis. Family characteristics of the patients with an MMR germline mutation were compared with those of patients with an unexplained microsatellite instable tumour.

## MATERIALS AND METHODS

### Patients

We examined 614 families, who visited the Department of Human Genetics of the Radboud University Nijmegen Medical Centre between 1997 and November 2005 because of possible familial colorectal cancer. Families were included because they either fulfilled the Amsterdam II criteria (Vasen *et al*, 1999) (*n*=126), or fulfilled the Bethesda guidelines (Rodriguez-Bigas *et al*, 1997) (*n*=333), or had a history very close to the Bethesda guidelines (*n*=155). Such patients are suspected of Lynch syndrome, which is defined as cancer owing to a germline mutation in one of the MMR genes (Jass, 2006).

Two diagnostic molecular strategies were used. Mutation analysis of germline DNA was performed as the first test in 83 families, who fulfilled clinical criteria for Lynch syndrome, or in which no tumour material was available. MSI analysis was also performed in 43 of these families. In the other 531 families MSI analysis was used as the initial step to select patients for germline mutation analysis.

The index tumour of a family was defined as the MSI-positive tumour that was diagnosed at the youngest age or, in case MSI analysis was not performed, the first tumour of the patient in whom the germline mutation was detected. The study was performed in accordance with the rules of the Radboud University Nijmegen Medical Centre Medical Ethical Committee.

### Germline mutation analysis of the MMR system

Mutation analysis of *MLH1*, *PMS2*, *MSH2*, and *MSH6* was performed in DNA from peripheral blood lymphocytes by a combination of either single-strand conformation polymorphism analysis or denaturing gradient gel electrophoresis and direct sequence analysis essentially as described elsewhere (Wu *et al*, 1997; Hoogerbrugge *et al*, 2003). Only mutations resulting in a premature termination codon, the recurrent amino-acid deletion in *MLH1* c.1852_1854del (p.Lys618del) and the amino-acid deletion c.211_213del (p.Glu71del) in *MLH1*, which has been shown to abort the function of MLH1 (Raevaara *et al*, 2002), were considered pathogenic. For the detection of large deletions and duplications in *MLH1*, *MSH2*, *MSH6*, and *PMS2*, the P003 and P008 Multiplex Ligation-dependent Probe Amplification (MLPA) kits of MRC Holland (Amsterdam, the Netherlands) were used. All deletions and duplications were confirmed by Southern blot analysis essentially as described elsewhere (Wijnen *et al*, 1998) or with a specific PCR using primers flanking the deletion or one of the breakpoints of a duplicated region.

### MSI analysis

In total, 667 tumours in 574 families were tested for MSI: 566 colorectal carcinomas, 34 colorectal adenomas, 47 endometrium carcinomas, eight duodenum/small bowel/appendix carcinomas, one sebaceous carcinoma and one other skin tumour (trichoepithelioma or trichoblastoma), one ovarian carcinoma, and nine urothelial cell carcinomas.

MSI analysis was performed using the Bethesda panel of microsatellite markers (*D2S123*, *D5S346*, *D17S250*, *BAT25*, *BAT26*) (Boland *et al*, 1998). Tumours were scored as MSI positive if at least two of the five Bethesda markers showed instability; they were scored MSI negative if none of the Bethesda markers showed instability. In case of one instable marker, additional markers were included, and immunohistochemistry (IHC) of MMR proteins was performed (Hoogerbrugge *et al*, 2003). In 273 tumours, the mononucleotide marker *BAT40* was added to the standard set of markers and in 558 of 667 tumours IHC of MSH6 was performed irrespective of the MSI status to minimise the chance of missing a tumour that was due to an *MSH6* germline mutation.

### Immunohistochemistry

IHC was performed on formalin-fixed, paraffin-embedded tissues. Slides were stained with antibodies against MLH1 (Pharmingen code: 51-1327gr), PMS2 (Pharmingen code: 556415), MSH2 (Oncogene Research Products code: NA26), and MSH6 (Transduction Laboratories code: G70220). Staining patterns of MMR proteins were evaluated using normal epithelial, stromal, and inflammatory cells as internal controls. Stained slides were scored as (1) positive, that is showing nuclear staining in at least some tumour cells; (2) negative, that is no staining of the tumour with a positive internal control; or (3) not assessable, that is when the technical quality was insufficient to provide an unambiguous result despite repeated assays (de Jong *et al*, 2004).

### Analysis of hypermethylation of the *MLH1* promoter

The DNA methylation status of the *MLH1* promoter region was determined after bisulphite treatment of the DNA using the EZ DNA methylation KIT™, ZYMO Research. To avoid conversion of methylated cytosines to uracil, the modification time was optimised. Modification was performed in duplicate for 3 and 6 h, respectively. Methylation of the region of 337–154 bp upstream of the translational start site, which has been shown to correlate with *MLH1* expression, was analysed (Deng *et al*, 2002). FAM-labelled PCR products were generated using primers (5′-TATTTTTGTTTTTATTGGTTGGATA-3′ and 5′-AATACCAATCAAATTTCTCAACTCT-3′) flanking 11 CpG sites and analysed on an ABI PRISM 3730 Genetic Analyzer under denaturing conditions using genemapper software. The products of unmethylated and methylated DNA migrate at 186 and 183 bp, respectively. This was verified by digestion by *BstU*I (New England Biolabs, Beverly, MA, USA), which cleaves only the CGCG sequence, which is not converted by bisulphite treatment when methylated. To assess the amount of methylation, the peak height of the product at 183 bp was divided by the sum of the peak heights at 183 and 186 bp. The resulting percentage of methylation was corrected for the percentage of tumour cells. Most tumours with methylation of the *MLH1* promoter showed percentage of methylation above 80%, whereas in none of the adjacent normal tissues methylation was detected.

### Patient characteristics and pedigree analysis

The following information was obtained for all families as part of the genetic counselling procedure: age at diagnosis, type of cancer, number of family members who had cancer, their age at diagnosis, their type of cancer, and their relation with the patient. Pathological and surgical reports were evaluated whenever possible.

Pedigrees were scored as fulfilling the Amsterdam I criteria (Vasen *et al*, 1991), the Amsterdam II criteria (Vasen *et al*, 1999), or the criterion described by Rodriguez-Bigas *et al* (1997) that is: two first degree relatives with a cancer associated with Lynch syndrome, one of them with an age at diagnosis below 50 years. As this study was mainly directed at MSI in tumours, a positive score for fulfilment of one of these three criteria was only given if the index patient was part of the criterion. The occurrence of metachronous or synchronous cancers associated with Lynch syndrome was noted.

### Data analysis

Descriptive statistics were used to describe the results of the molecular laboratory tests, germline mutation analysis, and pedigree analysis. Categorical variables were checked for statistically significant differences using either the *χ*^2^ test or logistic regression. Continuous variables were checked for statistically significant differences using either the Student's *t*-test or analysis of variance. The Tukey–Kramer test was used to calculate differences in continuous variables between two groups with adjustment for multiple testing. *P*-values <0.05 were considered statistically significant. Analyses were performed with the SAS system for Windows V8.2.

## RESULTS

### Identification and characterisation of families with a germline MMR gene mutation

**Figure 1:**
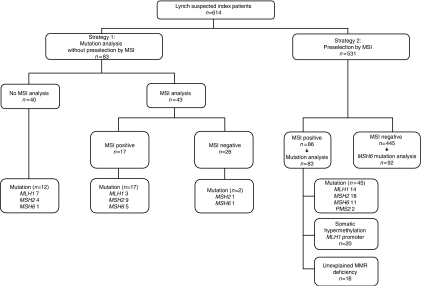
Analytic strategy of the study and number of patients in each analysis and for each result.

Our strategy 1 involved germline mutation analysis without prior testing for MSI in those families that fulfilled clinical criteria for Lynch syndrome, and those for whom no tumour DNA was available for MSI analysis. We found a germline mutation in 31 out of these 83 families (Figure 1, Table 1). In total, 43 index patients were tested for MSI within this group. MSI was detected in tumours of 17/43 patients. In all 17 MSI-positive index patients, a germline mutation in *MLH1*, *MSH2*, or *MSH6* was identified. One *MSH2* mutation was detected in a patient with an endometrial tumour diagnosed at age 39 that was tested MSI negative without loss of MLH1, PMS2, MSH2, or MSH6 protein staining. No other tumours from this family were available. One *MSH6* mutation was detected in a patient in whom a rectum tumour at an age of 51 years was MSI negative without loss of MLH1, PMS2, MSH2, or MSH6 protein staining. Material from a sigmoid carcinoma that occurred in another family member at the age of 33 years was not available.

Strategy 2 involved 531 families clinically suspected of Lynch syndrome. Here, a positive MSI test result was used to select families for germline mutation analysis. In 86/531 families, at least one MSI-positive tumour was detected. Although their tumours were MSI positive, three patients declined testing for germline mutations and for *MLH1* promoter methylation in their tumour. In the remaining 83 families, 45 MMR gene germline mutations were detected (Table 1).

Thus, a pathogenic germline mutation was found in 76 out of 614 families (12%) with a clinical history suggestive of Lynch syndrome. There were 24 mutations in *MLH1*, two in *PMS2*, 32 in *MSH2*, and 18 in *MSH6*.

Most MSI-positive tumours were also tested by IHC. Tumour cells of *MLH1* mutation carriers generally lacked MLH1 and PMS2 protein by IHC staining. Those of *MSH2* mutation carriers lacked MSH2 and MSH6. Tumours of *MSH6* mutation carriers lacked MSH6, and those of *PMS2* mutation carriers lacked PMS2. The IHC pattern correctly pinpointed the mutated gene in 50 of the 53 tumours (94%) where IHC was sufficiently informative.

We tested whether *MLH1* promoter methylation occurs in the presence of a germline mutation in one of the MMR genes. This was found to be a rare event. We tested a total of 42 microsatellite instable tumours from families with a germline mutation in *MLH1* (13), *PMS2* (1), *MSH2* (14), or *MSH6* (14). In only one of these tumours (a tumour with an *MSH6* germline mutation and absence of MSH6 protein staining, but presence of MLH1 protein staining), we detected incomplete *MLH1* promoter methylation (about 60%). Table 1 presents the results of analysis of *MLH1* promoter hypermethylation of index patients.

The majority (66%) of the proven Lynch syndrome families (50/76) fulfilled the Amsterdam II criteria. This was true for both strategy 1 (87%) and for strategy 2 (51%). The mean age at diagnosis of the index patients was 44 years (42 and 46 years in strategy 1 and 2, respectively).

### Identification and characterisation of families with hypermethylation of the *MLH1* promoter in their tumour

Pathogenic germline mutations in the MMR genes could not be detected in 38 families with at least one MSI-positive tumour (Figure 1). We therefore examined the methylation status of the *MLH1* promoter in 42 MSI-positive tumours of these 38 families. Methylation of the *MLH1* promoter was detected in 22 tumours (20 families) (Table 2). In the corresponding normal tissues *MLH1* promoter methylation was never detected, suggesting that the promoter methylation was not present in the germline.

In the group of 20 index patients with tumours with *MLH1* promoter methylation, we performed subsequent molecular analyses of other tumours of index patients or family members. Tumours either were MSI negative or showed methylation of the *MLH1* promoter in combination with absence of MLH1 protein staining (Table 2).

The Amsterdam II criteria were met in 11 of the 20 families (55%) with an MSI-positive tumour that was due to somatic hypermethylation of the *MLH1* promoter. The mean age at diagnosis of these index patients was 61 years.

### Characterisation of families with an unexplained MSI-positive tumour

There were 18 families with MSI-positive tumours, where neither a pathogenic MMR gene mutation was present, nor could hypermethylation of the *MLH1* promoter be demonstrated in the tumour (Table 3). These tumours were truly MSI positive, as in 17 of these tumours more than 75% of markers were instable, whereas the remaining tumour (no. 421) had three instable mononucleotide and three stable dinucleotide markers.

In 16 of the 18 index patients without a detectable germline mutation in an MMR gene and without hypermethylation of the *MLH1* promoter, the staining of at least one MMR protein was absent. The IHC patterns were in line with inactivation of *MLH1* (five families), *PMS2* (two families), *MSH2* (six families), and *MSH6* (one family (no. 421)). In the last family, an unclassified *MSH6* variant was detected for which the pathogenic nature could not be established (Kets *et al*, 2006). In two families, there was simultaneous loss of IHC protein staining of MSH2 and MSH6, and of PMS2.

Subsequent molecular analyses of tumours of family members of these index patients are presented in Table 3. Two index patients with absent *MSH2* staining had a family member with a tumour with an IHC pattern that also matched with *MSH2* inactivation, that is absence of MSH2 and MSH6.

Only two of the 18 families (11%) with an unexplained MSI-positive tumour fulfilled the Amsterdam II criteria. The mean age at diagnosis of these index patients was 44 years.

### Sensitivity of current mutation detection techniques

Mutation analysis was performed in 100 families with at least one tumour with MSI. In 20 of these families, *MLH1* promoter methylation was present. A pathogenic germline mutation was detected in 62 of the remaining 80 families (78%). Mostly, their clinical characteristics matched those of classical Lynch syndrome: a germline mutation was detected in 36 of 38 (95%) of those families that fulfilled the Amsterdam II criteria. Likewise, an MMR gene mutation was found in 47 of 54 (87%) of the families that met the following criterion of the Bethesda guidelines: two first degree relatives (including the index patient) with a tumour associated with Lynch syndrome, of which at least one was diagnosed below the age of 50 years.

### Comparison of characteristics of families with and without a detectable MMR gene mutation

The majority of families with an MMR gene mutation fulfilled the Amsterdam II criteria. In the 83 families selected by MSI only (strategy 2), we found that the clinical Amsterdam II criteria were met in 51% of the families in which an MMR gene mutation was eventually found compared with 11% of those in whom no such mutation was detected (*P*<0.009). The mean age at onset of the index patients was comparable in both groups (46 and 44 years, respectively). The mean age at diagnosis of index patients with a tumour with somatic hypermethylation of the *MLH1* promoter (61 years) was significantly higher than that of mutation positive index patients (Table 4).

## DISCUSSION

A disease causing germline mutation was identified in 78% of patients suspected of Lynch syndrome with an MSI-positive tumour and absence of hypermethylation of the *MLH1* promoter. Interestingly, the remaining 22% of patients with an unexplained MSI-positive tumour had a less pronounced family history of cancer, but were diagnosed at an age comparable with that of proven Lynch syndrome patients. Assessment bias is not likely to cause this difference, as the criteria for family history of cancer only include data from close relatives that should be known by the index patients. The large majority of the tumours in this group without gene mutations or promoter methylation, nonetheless showed loss of IHC staining of at least one of the MMR proteins. This is highly suggestive for the presence of either a germline mutation or of a somatic inactivation of the gene involved. These families had a much less prominent history of cancer, which might suggest the presence of a different type of mutation with a lower risk of cancer for relatives. Our current mutation detection protocol, which was highly effective in the classical Lynch syndrome families, did not pick up such putative mutations in this cohort.

The putative mutations might include (1) point mutations in the *PMS2* gene that are known to have a lower cancer risk (Truninger *et al*, 2005; Worthley *et al*, 2005; Hendriks *et al*, 2006); (2) germline methylation of the *MSH2* promoter as was recently described (Chan *et al*, 2006); (3) mutations in regulatory sequences or missence variants that might lead to a lower risk of tumour development in relatives because the complete inactivation of the affected MMR gene might be more dependent on modifier genes; and (4) a type of mutation that frequently arises *de novo*.

To our knowledge, this is the first study in which the difference in family history between patients with unexplained MSI-positive tumours and patients with a recognised germline mutation is addressed. The underlying mechanism for the familial occurrence of a tumour with MSI in such families remains unknown. Irrespective of this, confirmation for these findings in future studies might suggest that the clinical management in these families needs to be modified. For the time being, our surveillance advice for patients with an unexplained MSI-positive tumour and their close relatives remains identical to that of patients with Lynch syndrome including the start of surveillance at an early age.

The present study shows that the currently used techniques to detect a germline mutation have a sensitivity of 78% for patients with an MSI-positive tumour without hypermethylation of the *MLH1* promoter. A similar percentage can be calculated from data published by Hampel *et al* (2005): after exclusion of MSI-positive tumours with methylation of the *MLH1* promoter, they found a germline mutation in 23 out of 29 patients (79%) with an MSI-positive tumour. Their methods to analyse germline mutations are comparable with those used in our study. The sensitivity of germline mutation detection could not be deduced from other studies, as they did not include analysis of hypermethylation of the *MLH1* promoter and/or performed less comprehensive germline mutation analyses (Mangold *et al*, 2005; Barnetson *et al*, 2006; Niessen *et al*, 2006). Wagner *et al* (2003) also found a difference in prevalence of pathogenic *MLH1*, *MSH2*, or *MSH6* mutations between families that fulfilled the Amsterdam criteria (39 mutations in 49 families (80%)) and those not fulfilling these criteria (five mutations in 10 families (50%)) using methods that detect a similar type of mutations as the methods used in the present study. However, in this study data about the MMR deficiency of the tumours are missing.

In conclusion, this is the first study showing that almost all highly penetrant MMR gene mutations are identified with the currently used germline mutation detection techniques. The sensitivity of the currently used germline mutation detection techniques is at least 78% and probably near 100% in Lynch syndrome families with a highly penetrant mutation. A minority of MSI-positive tumours may be due to germline mutations in MMR genes that cannot yet be detected. Such putative mutations may confer a lower risk of cancers associated with Lynch syndrome for relatives.

## Figures and Tables

**Table 1:** Molecular laboratory tests results and patient characteristics of 76 patients with a pathogenic germline mutation in *MLH1*, *PMS2*, *MSH2*, *or MSH6*

**No.**	**S**	**Germline mutation**	**MSI**	**Meth**	**IHC**	**Tumour tested for MSI**	**Age**	**Other tumour(s)**	**AC**	**2fam**
*MLH1*										
8	1	c.15_28del (p.Gly6fs)	NT	NT	NT		42	co42,co47	+	+
288	2	c.18_34del (p.Val7fs)	+	−	MLH1/PMS2−	Caecum	40	−	−	−
189	2	c.211_213del (p.Glu71del)	+	−	PMS2/MSH6−	Endometrium	39	−	−	−
118	1	c.299C>T (p.Arg100X)	NT	NT	NT		26	co26,co37	+	+
645	2	c.578C>G (p.Ser193X)	+	−	MLH1/PMS2−	Caecum	26	co37,co39	−	+
163	1	c.588+3_588+6del (affects ss)	NT	NT	NT		50	co50,co50,co50	+	+
198	2	c.677G>A (p.Arg226Gln) (affects ss)	+	−	MLH1/PMS2−	Colon descendens	41	−	+	+
318	2	c.806C>G (p.Ser269X)	+	−	MLH1/PMS2−	Colon transversum	59	en49,co27	−	+
19	1	c.806C>G (p.Ser269X)	NT	NT	NT		45	co45	+	+
345	2	c.1225C>T (p.Gln409X)	+	−	MLH1/PMS2−	Sigmoid	44	−	+	+
454	2	c.1354del (p.Thr452fs)	+	−	MLH1/PMS2−	Colon transversum	49	−	+	+
335	2	c.1549G>T (p.Gly517X)	+	−	MLH1/PMS2−	Sigmoid	49	−	+	+
734	2	c.1852_1854del (p.Lys618del)	+	NT	MLH1/PMS2−	Urothelial cell carcinoma	60	co40,en54	−	+
428	2	c.1852_1854del (p.Lys618del)	+	NT	MLH1/PMS2−	Caecum	50		+	+
15	1	c.1852_1854del (p.Lys618del)	+	NT	MLH1/PMS2−	Endometrium	52	ov52,co52	−	+
451	2	c.1852_1854del (p.Lys618del)^a^	+	−	MLH1na/PMS2−	Colon ascendens	27	−	−	−
168	2	c.1852_1854del (p.Lys618del)	+	NT	NT	Caecum	42	−	+	+
42	1	c.1852_1854del (p.Lys618del)	NT	NT	NT		36	co36	+	+
3	1	c.1852_1854del (p.Lys618del)	+	NT	NT	Colon NOS	32	−	+	+
324	2	c.2103+1G>A	+	−	MLH1/PMS2−	Flexura linealis	50	co50	+	+
392	2	c.2103+1G>A	+	−	MLH1/PMS2−	Colon ascendens	38	−	+	+
65	1	c.2103+1G>A	+	−	MLH1/PMS2−	Rectum	42	−	+	+
222	1	c.2103+1G>A	NT	NT	NT		38	co38	+	+
260	1	c.2103+1G>A	NT	NT	NT		55	co55,en61	+	+
										
*PMS2*										
386	2	entire gene deletion	+	−	PMS2−	Colon ascendens	36	−	−	−
641	2	c.989–296_1144+706del	+	NT	PMS2−	Trichoepithelioma/trichoblastoma	45	−	−	−
										
*MSH2*										
322	2	c.1-?_211+?del	+	NT	MSH2/MSH6−	Colon ascendens	58	ur49	+	+
92	1	c.1-?_211+?del	+	NT	MSH2/MSH6−	Endometrium	41	−	+	+
5	1	c.1-?_211+?del	+	NT	NT		38	co42	+	+
614	2	c.1-?_366+?del	+	NT	MSH2/MSH6−	Flexura lienalis	21	−	−	−
730	2	c.1-?_1076+?del	+	NT	MSH2/MSH6−	Urothelial cell carcinoma	57	co42	+	+
528	2	c.1-?_1076+?del^b^	+	NT	none−	Caecum	32	en44	−	+
139	1	c.1-?_1076+?del	+	NT	MSH2/MSH6−	Endometrium	41	co42	+	+
287	2	c.1-?_1276+?del	+	−	MSH2/MSH6−	Caecum	33	−	+	+
700	2	c.212–1G>A	+	NT	MSH2-/MSH6na	Caecum	52	co61	−	+
350	2	c.212-?_366+?del	+	−	MSH6−	Caecum	72	−	+	+
117	1	c.212-?_366+?del	+	−	MSH2/MSH6−	Ileocecum	48	−	+	+
73	1	c.212-?_366+?del	+	NT	NT	Colon ascendens	44	en37,ov37	+	+
237	2	c.255dup (p.Glu86X)	+	NT	MSH2/MSH6−	Sebaceuous gland carcinoma	39	co25	+	+
190	2	c.367-?_645+?del	+	−	MSH2/MSH6−	Caecum	50	co50,co50	−	−
210	2	c.367-?_645+?del	+	−	MSH2/MSH6−	Colon transversum	43	−	+	+
366	2	c.642_645del (p.Gln215X)	+	NT	MSH2/MSH6−	Flexura linealis	46	−	+	+
18	1	c.793-?_1076+?del	+	−	MSH2/MSH6−	Rectum	29	co29	+	+
25	1	c.836del (p.Leu279fs)^c^	+	−	MSH2/MSH6−	Flexura linealis	46	−	+	+
37	1	c.862C>T (p.Gln288X)	NT	NT	NT		26	co26	+	+
196	2	c.915_922dup (p.Arg308fs)	+	NT	MSH2/MSH6−	Sigmoid	46	co46	−	−
11	1	c.943-?_1076+?del	+	−	MSH2/MSH6−	Endometrium	45	−	+	+
14	1	c.1147C>T (p.Arg383X)	NT	NT	NT		33	en33,co50	+	+
497	2	c.1165C>T (p.Arg389X)	+	NT	MSH2−/MSH6na	Flexura lienalis	44	−	−	−
462	2	c.1203dup (p.Gln402fs)	+	−	MSH2/MSH6−	Sigmoid	37	−	+	+
74	1	c.1203dup (p.Gln402fs)	+	−	MSH2−/MSH6na	Rectum	32	−	+	+
654	1	c.1255C>T (p.Gln419X)	NT	NT	NT		45	co45,co63	+	+
301	2	c.1277–2A>G	+	NT	MSH2/MSH6−	Caecum	33	−	−	+
637	2	c.1386+1G>T	+	−	MSH2/MSH6−	Colon transversum	47	co42	−	+
625	2	c.1387-?_1510+?del	+	−	MSH2/MSH6−	Endometrium	45	co53	+	+
107	1	c.1494dup (p.Ala499fs)	NT	NT	NT		46	co46	+	+
327	2	c.1861C>T (p.Arg621X)	+	NT	MSH2/MSH6−	Sigmoid	58	−	−	−
480	1	c.2005+1G>C	−	NT	none−	Endometrium	39	co45	+	+
										
*MSH6*										
206	1	c.1-?_457+?del^d^	+	NT	MSH6−	Urothelial cell carcinoma	56	ur57	−	+
657	2	c.261-?_457+?dup^d^	+	NT	MSH6−	Colon descendens	42	−	+	+
745	2	c.467C>G (p.Ser156X)	+	−	MSH6−	Ileocecum	63	−	+	+
138	2	c.651dup (p.Lys218X)^d^	+	−	MSH6−	Sigmoid	52	en37	+	+
342	1	c.814G>T (p.Glu272X)^d^,^f^	+	−	MSH6−	Endometrium	57	−	+	+
338	2	c.1135_1139del (p.Arg379X)^d^	+	−	MSH6−	Endometrium	38	ov38	−	−
137	1	c.1784del (p.Leu595fs)	−	NT	none−	Rectum	51		+	+
515	2	c.2815C>T (p.Gln939X)^a^,^d^	+	NT	MSH6−	Iieum	65	co38,co51,co58,ur69	−	+
105	1	c.3261del (p.Phe1088fs)^d^	+	−	MSH6−	Colon ascendens	39	−	+	+
766	2	c.3261del (p.Phe1088fs)	+	−	MSH6−	Endometrium	41	−	−	−
446	2	c.3261dup (p.Phe1088fs)^d^	+	−	MSH2/MSH6−	Endometrium	43	−	+	+
450	2	c.3273dup (p.Lys1092X)^d^	+	−	MSH6−	Colon ascendens	50	co46,co50	+	+
711	2	c.3438+1G>A	+	NT	MSH6na	Rectum	45	−	+	+
692	2	c.3438+1G>A	+	NT	MSH6na	Colon transversum	43	en53,ov43	−	−
500	2	c.3514dup (p.Arg1172fs)	+	+	MSH6−	Colon transversum	70	ur70	−	−
434	1	c.3678_3706dup (p.Ala1236fs)^d^	NT	NT	NT		38	en38	−	+
128	1	c.3838C>T (p.Gln1280X)^d^	+	−	MSH6−	Endometrium	36	−	+	+
886	1	c.4001G>A (p.Arg1334Gln) affects ss	+	NT	MSH6na^e^	Colon NOS	44	co61: MSI pos,IHC MSH6 NA	−	+

S, strategy of molecular testing; MSI, microsatellite instability; Meth, methylation analysis of *MLH1* promoter; IHC, immunohistochemical analysis of MLH1, PMS2, MSH2, and MSH6; Tumour tested for MSI, tumour origin or exact location of tumour in case of colon cancer of tumour tested for MSI; Age, age at diagnosis of tumour tested for MSI or age at diagnosis of (first) tumour in case MSI analysis was not performed; Other tumour(s), metachronous or synchronous cancer associated with Lynch syndrome of index patient who had MSI analysis or tumour(s) of index patient who did not have MSI analysis and age at diagnosis; AC, Amsterdam II criteria (Vasen *et al*, 1999); 2fam, 2 first degree relatives (including index) with cancer associated with Lynch syndrome, one of them with an age at diagnose below 50 years (Rodriguez-Bigas *et al*, 1997); 1, strategy 1 (germline mutation analysis without preselection by MSI analysis); 2, strategy 2 (first MSI analysis); ss, splice site; +, positive; −, negative; NT, not tested; NA, not assessable; NOS, not otherwise specified; co, colon; en, endometrium; ur, urothelial; ov, ovarian.

a Carrier status of patient deduced from mutation status of relatives.

b Also carrier of variant c.1A>G (p.Met1?) in *MSH2* (paper in preparation).

c Also carrier of variant c.965G>A(p.Gly322Asp) in *MSH2*.

d Mutations published elsewhere (Kets *et al*, 2006).

e Adenocarcinoma Caecum of sister with same mutation MSI and IHC MSH6− (1co46 MSI IHC MSH6−).

f Also carrier of variant c.65G>C (p.Gly22Ala) in *MLH1*.

**Table 2:** Molecular laboratory tests results and family history of 20 patients with an MSI-positive tumour with somatic *MLH1* promoter methylation

**No.**	**Meth**	**IHC**	**Tumour tested for MSI**	**Age**	**Other tumour(s)**	**AC**	**2fam**	**Tumours of close relatives**
312	+	MLH1/PMS2−	Caecum	63	−	+	+	1co53 MSS
408	+	MLH1/PMS2−	Colon ascendens	62	co40	+	+	
642^a^	+	MLH1/PMS2−	Colon transversum	62	co62: MSI, IHC MLH1−, meth+	−	−	1co75 MSI,IHC: MLH1−,meth+
477	+	MLH1/PMS2−	Colon transversum	75	co75	−	−	2co50 MSS
226	+	MLH1/PMS2−	Colon ascendens	67	−	+	+	
755	+	MLH1/PMS2−	Colorectum NOS	72	co72	−	−	
299	+	MLH1/PMS2−	Colon ascendens	50	co50	+	−	1co59 MSS
154	+	MLH1/PMS2−	Colon ascendens	71	−	+	+	1co38 MSS
771	+	MLH1/PMS2−	Colon ascendens	65	co65	−	−	
683	+	PMS2−	Colon ascendens	69	−	−	−	1co58 MSS
785	+	MLH1/PMS2−	Colon descendens	54	co54	−	−	
142	+	MLH1/PMS2−	Colon ascendens	55	en57	+	+	1co22 MSS
86	+	MLH1/PMS2−	Colon ascendens	55	−	+	+	
482^b^	+	MLH1/PMS2−	Caecum	65	co64	+	−	
748	+	MLH1/PMS2−	Colorectum NOS	45	−	−	−	
57	+	MLH1/PMS2−	Colon transversum	49	−	+	+	
441	+	MLH1/PMS2−	Caecum	51	−	−	−	
316	+	MLH1/PMS2−	Colon NOS	71	en70 MSS,co71	+	+	2co43 MSS
331	+	MLH1/PMS2−	Duodenum	47	−	−	−	
655	+	MLH1/PMS2/MSH2/MSH6−	Colon descendens	64	co47 MSS, ur64 MSS	+	+	

Meth, methylation analysis of *MLH1* promoter; IHC, immunohistochemical analysis of MLH1, PMS2, MSH2, and MSH6; Tumour tested for MSI, tumour origin or exact location of tumour in case of colon cancer of tumour tested for MSI; Age, age at diagnosis of tumour tested for MSI; Other tumour(s), metachronous or synchronous cancer associated with Lynch syndrome of index patient and age at diagnosis; AC, Amsterdam II criteria (Vasen *et al*, 1999); 2fam, 2 first degree relatives (including index) with a Lynch syndrome associated cancer, one of them with an age at diagnose below 50 years (Rodriguez-Bigas *et al*, 1997); Tumours of close relatives, tumours of close relatives tested for MSI and/or IHC, meth; +, positive; −, negative; NOS, not otherwise specified; co, colon; en, endometrium; ur, urothelial; MSI, MSI positive; MSS, MSI negative; 1, first degree relative; 2, second degree relative. (e.g. 1co53 MSS, a first degree relative of index patient had a colon tumour diagnosed at the age of 53 years which was MSI negative).

a Carrier of unclassified variant c.3744_3773dup (p.His1248_Ser1257dup) in *MSH6*.

b Carrier of unclassified variant c.663A>C (p.Glu221Asp) in *MSH6.*

**Table 3:** Molecular laboratory tests results and family history of 18 patients with an MSI-positive tumour with unexplained etiology

**No.**	**Meth**	**IHC**	**Tumour tested for MSI**	**Age**	**Other tumour(s)**	**AC**	**2fam**	**Tumours of close relatives**
149^a^	−	MLH1/PMS2−	Colon ascendens	43	−	−	−	3co45 MSS
499	−	MLH1/PMS2−	Colon ascendens	58	−	−	−	
445	−	MLH1/PMS2−	Colon transversum	49	−	−	+	
498	−	MLH1/PMS2−	Sigmoid	58	co49	−	−	
172	−	MLH1/PMS2−	Colon ascendens	51	−	−	+	
373^a^	−	PMS2−	Caecum	36	−	−	−	
554	−	PMS2−	Colon transversum	55	co55	−	−	1co56 MSS
582	−	MSH2/MSH6−	Sigmoid	33	−	+	+	1co47 MSI,IHC:MSH2−,meth−
396	−	MSH2/MSH6−	Appendix	34	−	−	−	
224	−	MSH2/MSH6−	Ileocecum	53	co34,co50	+	+	
580	−	MSH2/MSH6−	Endometrium	45	−	−	−	
718	−	MSH2/MSH6−	Rectum	18	−	−	+	1co47 MSI,HC:MSH2−,meth−
736	−	MSH2/MSH6−	Rectum	58	co58 MSS	−	−	
421^b^	−	MSH6−	Colon ascendens	53	−	−	−	1en62 MSI/IHC NA
135	−	PMS2/MSH2/MSH6−c	Colon ascendens	27	−	−	−	
243	−	PMS2/MSH2/MSH6−c	Colon transversum	30	−	−	+	
127	−	None−	Rectum	54	−	−	−	
375^d^	−	None−	Caecum	36	−	−	+	

Meth, methylation analysis of *MLH1* promoter; IHC, immunohistochemical analysis of MLH1, PMS2, MSH2, and MSH6; Tumour tested for MSI, tumour origin or exact location of tumour in case of colon cancer of tumour tested for MSI; Age, age at diagnosis of tumour tested for MSI; Other tumour(s), metachronous or synchronous cancer associated with Lynch syndrome of index patient and age at diagnosis; AC, Amsterdam II criteria (Vasen *et al*, 1999). 2fam, 2 first degree relatives (including index) with Lynch syndrome associated cancer, one of them with an age at diagnose below 50 years (Rodriguez-Bigas *et al*, 1997); Tumours of close relatives, tumours of close relatives tested for MSI and/or IHC, meth; +, positive; −, negative; NA, not assessable; co, colon; MSI, MSI positive; MSS, MSI negative; 1, first degree relative; 2, second degree relative; 3 third degree relative. (e.g. 3co45 MSS, a third degree relative of index patient had a colon tumour diagnosed at the age of 45 years which was MSI negative).

a Carrier of unclassified variant c.1852_1853delinsGC (p.Lys618Ala) in *MLH1*.

b Carrier of unclassified variant c.2117T>C (p.Phe706Ser) in *MSH6* (Kets *et al*, 2006).

c IHC difficult to interpret.

d Carrier of unclassified variant c.250A>G (p.Lys84Glu) in *MLH1* and c.984C>T (silent) in *MSH2*.

**Table 4:** Family history and patient characteristics of index patients with an MSI-positive tumour who were preselected by MSI analysis (strategy 2): a comparison between patients with a germline mutation, with somatic methylation of the *MLH1* promoter and with a tumour with unexplained MSI

	**Germline mutation *MLH1*, *PMS2*, *MSH2*, or *MSH6* *N*=45**	**Somatic methylation of *MLH1* promoter *N*=20**	**Unexplained MMR deficiency *N*=18**	**Germline mutation *vs* somatic methylation (*P*-value)**	**Germline mutation *vs* unexplained MMR deficiency (*P*-value)**
Amsterdam I criteria positive^a^	16 (36%)	6 (30%)	2 (11%)	0.66	0.07
Amsterdam II criteria positive^a^	23 (51%)	11 (55%)	2 (11%)	0.77	**0.009**
Two first degree relatives with Lynch syndrome associated cancer, one below 50 years^a^	31 (69%)	9 (45%)	7 (39%)	0.07	**0.03**
Metachronous or synchronous Lynch syndrome associated cancers of index patient	17 (38%)	10 (50%)	4 (22%)	0.20	0.24
Age at diagnosis of MSI-positive index tumour	45.9 [42.6–49.1]	60.6 [55.7–65.5]	43.9 [38.8–49.1]	**<0.0001**	0.80
Age at diagnosis of first Lynch syndrome associated cancer of index patient	43.1 [39.9–46.3]	58.6 [53.8–63.4]	42.4 [37.3–47.5]	**<0.0001**	0.97
Mean age of two youngest relatives with Lynch syndrome associated cancer^b^	45.7 [40.3–51.1]	54.0 [46.3–61.7]	49.5 [31.5–67.5]	0.19	0.91

a A positive score for fulfilment was only given if the index patient was part of the criterion.

b Mean age of the two youngest affected relatives of the index patient. This was only calculated for families that fulfilled the Amsterdam II criteria.

Bold values signify *P*-values <0.05.

## References

[bib1] Aaltonen LA, Salovaara R, Kristo P, Canzian F, Hemminki A, Peltomaki P, Chadwick RB, Kaariainen H, Eskelinen M, Jarvinen H, Mecklin JP, de la Chapelle A (1998) Incidence of hereditary nonpolyposis colorectal cancer and the feasibility of molecular screening for the disease. N Engl J Med 338: 1481–1487 9593786 10.1056/NEJM199805213382101

[bib2] Barnetson RA, Tenesa A, Farrington SM, Nicholl ID, Cetnarskyj R, Porteous ME, Campbell H, Dunlop MG (2006) Identification and survival of carriers of mutations in DNA mismatch-repair genes in colon cancer. N Engl J Med 354: 2751–2763 16807412 10.1056/NEJMoa053493

[bib3] Boland CR, Thibodeau SN, Hamilton SR, Sidransky D, Eshleman JR, Burt RW, Meltzer SJ, Rodriguez-Bigas MA, Fodde R, Ranzani GN, Srivastava S (1998) A National Cancer Institute Workshop on Microsatellite Instability for cancer detection and familial predisposition: development of international criteria for the determination of microsatellite instability in colorectal cancer. Cancer Res 58: 5248–5257 9823339

[bib4] Chan TL, Yuen ST, Kong CK, Chan YW, Chan AS, Ng WF, Tsui WY, Lo MW, Tam WY, Li VS, Leung SY (2006) Heritable germline epimutation of MSH2 in a family with hereditary nonpolyposis colorectal cancer. Nat Genet 38: 1178–1183 16951683 10.1038/ng1866

[bib5] Cunningham JM, Kim CY, Christensen ER, Tester DJ, Parc Y, Burgart LJ, Halling KC, McDonnell SK, Schaid DJ, Walsh VC, Kubly V, Nelson H, Michels VV, Thibodeau SN (2001) The frequency of hereditary defective mismatch repair in a prospective series of unselected colorectal carcinomas. Am J Hum Genet 69: 780–790 11524701 10.1086/323658 PMC1226064

[bib6] de Jong AE, van Puijenbroek M, Hendriks Y, Tops C, Wijnen J, Ausems MG, Meijers-Heijboer H, Wagner A, van Os TA, Brocker-Vriends AH, Vasen HF, Morreau H (2004) Microsatellite instability, immunohistochemistry, and additional PMS2 staining in suspected hereditary nonpolyposis colorectal cancer. Clin Cancer Res 10: 972–980 14871975 10.1158/1078-0432.ccr-0956-3

[bib7] Deng G, Peng E, Gum J, Terdiman J, Sleisenger M, Kim YS (2002) Methylation of hMLH1 promoter correlates with the gene silencing with a region-specific manner in colorectal cancer. Br J Cancer 86: 574–579 11870540 10.1038/sj.bjc.6600148 PMC2375277

[bib8] Dove-Edwin I, de Jong AE, Adams J, Mesher D, Lipton L, Sasieni P, Vasen HF, Thomas HJ (2006) Prospective results of surveillance colonoscopy in dominant familial colorectal cancer with and without Lynch syndrome. Gastroenterology 130: 1995–2000 16762622 10.1053/j.gastro.2006.03.018

[bib9] Gazzoli I, Loda M, Garber J, Syngal S, Kolodner RD (2002) A hereditary nonpolyposis colorectal carcinoma case associated with hypermethylation of the MLH1 gene in normal tissue and loss of heterozygosity of the unmethylated allele in the resulting microsatellite instability-high tumor. Cancer Res 62: 3925–3928 12124320

[bib10] Hampel H, Frankel WL, Martin E, Arnold M, Khanduja K, Kuebler P, Nakagawa H, Sotamaa K, Prior TW, Westman J, Panescu J, Fix D, Lockman J, Comeras I, de la Chapelle A (2005) Screening for the Lynch syndrome (hereditary nonpolyposis colorectal cancer). N Engl J Med 352: 1851–1860 15872200 10.1056/NEJMoa043146

[bib11] Hendriks YM, Jagmohan-Changur S, van der Klift H, Morreau H, van Puijenbroek M, Tops C, van Os T, Wagner A, Ausems MG, Gomez E, Breuning MH, Brocker-Vriends AH, Vasen HF, Wijnen JT (2006) Heterozygous mutations in PMS2 cause hereditary nonpolyposis colorectal carcinoma (Lynch syndrome). Gastroenterology 130: 312–322 16472587 10.1053/j.gastro.2005.10.052

[bib12] Hitchins M, Williams R, Cheong K, Halani N, Lin VA, Packham D, Ku S, Buckle A, Hawkins N, Burn J, Gallinger S, Goldblatt J, Kirk J, Tomlinson I, Scott R, Spigelman A, Suter C, Martin D, Suthers G, Ward R (2005) MLH1 germline epimutations as a factor in hereditary nonpolyposis colorectal cancer. Gastroenterology 129: 1392–1399 16285940 10.1053/j.gastro.2005.09.003

[bib13] Hoogerbrugge N, Willems R, van Krieken JH, Kiemeney LA, Weijans M, Nagengast FM, Arts N, Brunner HG, Ligtenberg MJ (2003) Very low incidence of microsatellite instability in rectal cancers from families at risk for HNPCC. Clin Genet 63: 64–70 12519374 10.1034/j.1399-0004.2003.630110.x

[bib14] Jass JR (2006) Hereditary Non-Polyposis Colorectal Cancer: the rise and fall of a confusing term. World J Gastroenterol 12: 4943–4950 16937488 10.3748/wjg.v12.i31.4943 PMC4087395

[bib15] Kets CM, van Krieken JH, Hebeda KM, Wezenberg SJ, Goossens M, Brunner HG, Ligtenberg MJ, Hoogerbrugge N (2006) Very low prevalence of germline MSH6 mutations in hereditary non-polyposis colorectal cancer suspected patients with colorectal cancer without microsatellite instability. Br J Cancer 95: 1678–1682 17117178 10.1038/sj.bjc.6603478 PMC2360757

[bib16] Lindor NM, Rabe K, Petersen GM, Haile R, Casey G, Baron J, Gallinger S, Bapat B, Aronson M, Hopper J, Jass J, LeMarchand L, Grove J, Potter J, Newcomb P, Terdiman JP, Conrad P, Moslein G, Goldberg R, Ziogas A, Anton-Culver H, de AM, Siegmund K, Thibodeau SN, Boardman LA, Seminara D (2005) Lower cancer incidence in Amsterdam-I criteria families without mismatch repair deficiency: familial colorectal cancer type X. JAMA 293: 1979–1985 15855431 10.1001/jama.293.16.1979 PMC2933042

[bib17] Lynch HT, de la Chapelle A (2003) Hereditary colorectal cancer. N Engl J Med 348: 919–932 12621137 10.1056/NEJMra012242

[bib18] Mangold E, Pagenstecher C, Friedl W, Mathiak M, Buettner R, Engel C, Loeffler M, Holinski-Feder E, Muller-Koch Y, Keller G, Schackert HK, Kruger S, Goecke T, Moeslein G, Kloor M, Gebert J, Kunstmann E, Schulmann K, Ruschoff J, Propping P (2005) Spectrum and frequencies of mutations in MSH2 and MLH1 identified in 1,721 German families suspected of hereditary nonpolyposis colorectal cancer. Int J Cancer 116: 692–702 15849733 10.1002/ijc.20863

[bib19] Miyakura Y, Sugano K, Akasu T, Yoshida T, Maekawa M, Saitoh S, Sasaki H, Nomizu T, Konishi F, Fujita S, Moriya Y, Nagai H (2004) Extensive but hemiallelic methylation of the hMLH1 promoter region in early-onset sporadic colon cancers with microsatellite instability. Clin Gastroenterol Hepatol 2: 147–156 15017620 10.1016/s1542-3565(03)00314-8

[bib20] Niessen RC, Berends MJ, Wu Y, Sijmons RH, Hollema H, Ligtenberg MJ, de Walle HE, de Vries EG, Karrenbeld A, Buys CH, van der Zee AG, Hofstra RM, Kleibeuker JH (2006) Identification of mismatch repair gene mutations in young colorectal cancer patients and patients with multiple HNPCC-associated tumours. Gut 55: 1781–1788 16636019 10.1136/gut.2005.090159 PMC1856475

[bib21] Raevaara TE, Timoharju T, Lonnqvist KE, Kariola R, Steinhoff M, Hofstra RM, Mangold E, Vos YJ, Nystrom-Lahti M (2002) Description and functional analysis of a novel in frame mutation linked to hereditary non-polyposis colorectal cancer. J Med Genet 39: 747–750 12362032 10.1136/jmg.39.10.747 PMC1734999

[bib22] Rodriguez-Bigas MA, Boland CR, Hamilton SR, Henson DE, Jass JR, Khan PM, Lynch H, Perucho M, Smyrk T, Sobin L, Srivastava S (1997) A National Cancer Institute Workshop on Hereditary Nonpolyposis Colorectal Cancer Syndrome: meeting highlights and Bethesda guidelines. J Natl Cancer Inst 89: 1758–1762 9392616 10.1093/jnci/89.23.1758

[bib23] Samowitz WS, Albertsen H, Herrick J, Levin TR, Sweeney C, Murtaugh MA, Wolff RK, Slattery ML (2005) Evaluation of a large, population-based sample supports a CpG island methylator phenotype in colon cancer. Gastroenterology 129: 837–845 16143123 10.1053/j.gastro.2005.06.020

[bib24] Suter CM, Martin DI, Ward RL (2004) Germline epimutation of MLH1 in individuals with multiple cancers. Nat Genet 36: 497–501 15064764 10.1038/ng1342

[bib25] Truninger K, Menigatti M, Luz J, Russell A, Haider R, Gebbers JO, Bannwart F, Yurtsever H, Neuweiler J, Riehle HM, Cattaruzza MS, Heinimann K, Schar P, Jiricny J, Marra G (2005) Immunohistochemical analysis reveals high frequency of PMS2 defects in colorectal cancer. Gastroenterology 128: 1160–1171 15887099 10.1053/j.gastro.2005.01.056

[bib26] Valle L, Carbonell P, Fernandez V, Dotor A, Sanz M, Benitez J, Urioste M (2007a) MLH1 germline epimutations in selected patients with early-onset non-polyposis colorectal cancer. Clin Genet 71: 232–237 17309645 10.1111/j.1399-0004.2007.00751.x

[bib27] Valle L, Perea J, Carbonell P, Fernandez V, Dotor AM, Benitez J, Urioste M (2007b) Clinicopathologic and pedigree differences in Amsterdam I-Positive Hereditary Nonpolyposis Colorectal Cancer families according to tumor microsatellite instability status. J Clin Oncol 25: 781–786 17228022 10.1200/JCO.2006.06.9781

[bib28] Vasen HF, Mecklin JP, Khan PM, Lynch HT (1991) The International Collaborative Group on Hereditary Non-Polyposis Colorectal Cancer (ICG-HNPCC). Dis Colon Rectum 34: 424–425 2022152 10.1007/BF02053699

[bib29] Vasen HF, Watson P, Mecklin JP, Lynch HT (1999) New clinical criteria for hereditary nonpolyposis colorectal cancer (HNPCC, Lynch syndrome) proposed by the International Collaborative group on HNPCC. Gastroenterology 116: 1453–1456 10348829 10.1016/s0016-5085(99)70510-x

[bib30] Wagner A, Barrows A, Wijnen JT, van der Klift H, Franken PF, Verkuijlen P, Nakagawa H, Geugien M, Jaghmohan-Changur S, Breukel C, Meijers-Heijboer H, Morreau H, van Puijenbroek M, Burn J, Coronel S, Kinarski Y, Okimoto R, Watson P, Lynch JF, de la Chapelle A, Lynch HT, Fodde R (2003) Molecular analysis of hereditary nonpolyposis colorectal cancer in the United States: high mutation detection rate among clinically selected families and characterization of an American founder genomic deletion of the MSH2 gene. Am J Hum Genet 72: 1088–1100 12658575 10.1086/373963 PMC1180263

[bib31] Weisenberger DJ, Siegmund KD, Campan M, Young J, Long TI, Faasse MA, Kang GH, Widschwendter M, Weener D, Buchanan D, Koh H, Simms L, Barker M, Leggett B, Levine J, Kim M, French AJ, Thibodeau SN, Jass J, Haile R, Laird PW (2006) CpG island methylator phenotype underlies sporadic microsatellite instability and is tightly associated with BRAF mutation in colorectal cancer. Nat Genet 38: 787–793 16804544 10.1038/ng1834

[bib32] Wijnen J, van der Klift H, Vasen H, Khan PM, Menko F, Tops C, Meijers-Heijboer H, Lindhout D, Moller P, Fodde R (1998) MSH2 genomic deletions are a frequent cause of HNPCC. Nat Genet 20: 326–328 9843200 10.1038/3795

[bib33] Worthley DL, Walsh MD, Barker M, Ruszkiewicz A, Bennett G, Phillips K, Suthers G (2005) Familial mutations in PMS2 can cause autosomal dominant hereditary nonpolyposis colorectal cancer. Gastroenterology 128: 1431–1436 15887124 10.1053/j.gastro.2005.04.008

[bib34] Wu Y, Nystrom-Lahti M, Osinga J, Looman MW, Peltomaki P, Aaltonen LA, de la Chapelle A, Hofstra RM, Buys CH (1997) MSH2 and MLH1 mutations in sporadic replication error-positive colorectal carcinoma as assessed by two-dimensional DNA electrophoresis. Genes Chromosomes Cancer 18: 269–278 9087566

